# Whole-Body Adaptive Functional Electrical Stimulation Kinesitherapy Can Promote the Restoring of Physiological Muscle Synergies for Neurological Patients

**DOI:** 10.3390/s22041443

**Published:** 2022-02-13

**Authors:** Alessandro Scano, Robert Mihai Mira, Guido Gabbrielli, Franco Molteni, Viktor Terekhov

**Affiliations:** 1 UOS STIIMA Lecco—Human-Centered, Smart & Safe, Living Environment, Italian National Research Council (CNR), Via Previati 1/E, 23900 Lecco, Italy; robertmihai.mira@stiima.cnr.it; 2 VIKTOR S.r.l.—Via Pasubio, 5, 24044 Dalmine (BG), Italy; guido.gabbrielli@gmail.com; 3 Villa Beretta Rehabilitation Center, Ospedale Valduce, Via N. Sauro 17, 23845 Costa Masnaga, Italy; franco56.molteni@gmail.com

**Keywords:** muscle synergies, whole body FES, neurological patients

## Abstract

Background: Neurological diseases and traumas are major factors that may reduce motor functionality. Functional electrical stimulation is a technique that helps regain motor function, assisting patients in daily life activities and in rehabilitation practices. In this study, we evaluated the efficacy of a treatment based on whole-body Adaptive Functional Electrical Stimulation Kinesitherapy (AFESK™) with the use of muscle synergies, a well-established method for evaluation of motor coordination. The evaluation is performed on retrospectively gathered data of neurological patients executing whole-body movements before and after AFESK-based treatments. Methods: Twenty-four chronic neurologic patients and 9 healthy subjects were recruited in this study. The patient group was further subdivided in 3 subgroups: hemiplegic, tetraplegic and paraplegic. All patients underwent two acquisition sessions: before treatment and after a FES based rehabilitation treatment at the VIKTOR Physio Lab. Patients followed whole-body exercise protocols tailored to their needs. The control group of healthy subjects performed all movements in a single session and provided reference data for evaluating patients’ performance. sEMG was recorded on relevant muscles and muscle synergies were extracted for each patient’s EMG data and then compared to the ones extracted from the healthy volunteers. To evaluate the effect of the treatment, the motricity index was measured and patients’ extracted synergies were compared to the control group before and after treatment. Results: After the treatment, patients’ motricity index increased for many of the screened body segments. Muscle synergies were more similar to those of healthy people. Globally, the normalized synergy similarity in respect to the control group was 0.50 before the treatment and 0.60 after (*p* < 0.001), with improvements for each subgroup of patients. Conclusions: AFESK treatment induced favorable changes in muscle activation patterns in chronic neurologic patients, partially restoring muscular patterns similar to healthy people. The evaluation of the synergic relationships of muscle activity when performing test exercises allows to assess the results of rehabilitation measures in patients with impaired locomotor functions.

## 1. Introduction

The aging of the population in the Western countries and the increased awareness of the economic and social costs of accidents at work are topical. In fact, it is estimated that in Europe about five million people [1] suffer from pathologies or have suffered trauma of varying severity to the neuro-muscular system. Furthermore, neural aging also leads to the development of various forms and degrees of motor impairment. In 2018, 19.7% of the EU population were 65 or older [2]. A need of advancements in the prevention and cure of neurologic illnesses clearly emerges. In this context, rehabilitation therapies can slow the effects of aging and help improve quality of life [3]. Other than being a physical and psychological burden to the individual, neurological diseases represent also a strain on the community, due to the need to provide aid to impaired individuals either by creating adequate structures for rehabilitation or providing healthcare. According to Eurostat, curative and rehabilitative therapies account for more than 50% of current health expenditure in most EU Member States [4].

In this context, the interest of scientists and practitioners in functional electrical stimulation for the rehabilitation of neurological patients with severe disorders of the musculoskeletal system has grown. Neuromuscular electric stimulation (NMES) has often been used to aid in the recovery of lost motor function [5,6,7,8]. The combined action of the patient’s neurostimulation and mobilization programs allows the brain to re-educate to recognize muscle stimuli as its own, triggering a series of nervous processes that favor the reactivation of impaired functional capacities (neuroplasticity) [9,10]. Through controlled and synchronized stimulation of specific areas of the body, physicists and therapists can provide functionality to muscle contractions. During the years, this specific branch of NMES has acquired the title of functional electrical stimulation (FES). Many studies have investigated the effects of FES on stroke survivors, in a variety of applications. In gait rehabilitation [11,12], increased stability, improved gait independence and higher gait speed were found after FES treatments. Functional electric stimulation has been also used in rehabilitation of the upper extremity in stroke survivors and allowed to achieve finer hand movements such and finger flexion [13], hand grasping [14] and broader arm movements [15]. In all these studies, the participants regained functionality of the upper extremity confirming the usefulness of FES. Furthermore, in [14] the authors compared the effects of basic electric muscle stimulation with EMG controlled FES and demonstrated that patients who underwent EMG controlled FES treatment performed better than patients who underwent basic electrical stimulation. FES has been also applied in gait rehabilitation for spinal cord injury patients proving its usefulness in aiding the rehabilitation process [16]. Other notable applications of FES have been in aiding full-face transplantation patients regain facial expressions [17]. Ultimately, FES allowed patients to retain functionality even while not using the devices in multiple scenarios [18].

Other studies employed FES for the recovery of upper extremity functionality with the aid of robotic instrumentation [19]. FES was also combined with complex control mechanisms like artificial neural networks trained to mimic natural muscle recruitment patterns, allowing impaired individuals to restore walking patterns [20].

Indeed, the modern view of human movement management is characterized by a multilevel hierarchical system between the brain and the muscular system [21]. These levels are anatomically and functionally connected and communicate through continuous feedback, in order to ensure movement regulation and correct motor performance. The repetition of motor gestures allows the improvement of the execution of the motor task [22,23,24]. It is known that if the activation of the muscle mass generated by the electrical impulse corresponds to the voluntary physiological activation [25], the brain recognizes stimuli as its own and automatically activates functions that tend to restore the connections that govern the part of the body affected by pathology or trauma and improve its functionality [26].

In rehabilitation scenarios, one of the most promising approaches for improving the prevention of diseases and prescriptions of treatments with novel data for clinicians is the decomposition of the electromyographic signal into muscle synergies [27]. The muscle synergy technique offers the possibility to analyze electromyographic recordings considering the natural couplings between muscles, and thus is a tool useful for the analysis of the modular organization of the human neuro-musculoskeletal system. Muscle synergies propose that the CNS relies on a limited number of modules [28], possibly implemented at the neural level [29], to simplify motion production. Consequently, by appropriately recruiting spatial modules with temporal activation coefficients, the CNS exploits a reduced set of preformed neural pathways, called synergies, to obtain a wide variety of motor outputs. Applications of muscle synergy included, among others, investigations on the muscle synergies of the upper limb in physiological conditions [30,31] and the effect of neurological injuries [32,33,34]. Synergies have also been applied to investigate locomotion [35,36,37,38] and postural control [39,40].

However, currently this evaluation approach has rarely been used to evaluate the efficacy of a rehabilitation program on subjects with CNS lesions based on electrical stimulation, and always on very limited number of subjects.

Given that muscle synergies have proven to be a useful tool to study muscle coordination patterns and that FES is considered a valuable technique to aid motor re-learning [41], it is natural for the two techniques to be used as complementary approaches [42]. In fact, some studies have already used both tools for robot guided rehabilitation [43] where muscle synergies are used to drive a functional electric stimulation system. Researchers have used FES and muscle synergies of healthy people to guide gait rehabilitation for post stroke patients [44] and to study the effects of a FES based rehabilitation technique on post stroke patients during cycling exercises [41]. In their studies, the authors found a significant improvement when comparing synergy similarity to healthy controls before and after the treatment.

In recent years total body electric stimulation (or whole-body electrostimulation) has become a valuable clinical practice [45]. This technique is the natural evolution of FES, it makes use of more electrodes and applies electric stimulation to a wider variety of muscles at once. It was reported that synchronizing the stimuli makes it possible to exercise complete kinetic chains with a synergistic approach guarantying more natural and fluid movements [46].

The overall improvements of whole-body electric stimulation come in the form of the ability to train a vaster array of possible movements and better implement motor control aids to impaired subjects. Another important feature generally observed in whole body electric stimulation is the co-contraction of agonist and antagonist muscles. Antagonist muscles can contribute to the improvement of aerobic strength without presenting damage to the motor patterns [47,48].

The growing interest of neurophysiology in clarifying the physiological mechanisms of the use of electrical stimulation for the treatment of locomotor dysfunctions is known [49]; however, few studies are available on assessing the effects of FES on neurologic patients with the use of muscle synergies when evaluating rehabilitation based on total-body movements. The aim of this study is to propose a pilot study for assessing the effects of a FES-based rehabilitation treatment on neurological patients. We aimed at showing that neuroplasticity can be induced and physiological muscle synergies can be partially restored in chronic neurological patients after a FES-based treatment in patients with various pathologies.

## 2. Materials and Methods

### 2.1. Participants

Twenty-four patients were recruited in this study. The included patients were divided into three groups: 8 with hemiplegia/paresis patients; 8 with paraplegia/paresis patients; 8 with tetraplegia/paresis patients. All patients were in the chronic stage of their disease. A control group composed of 9 healthy individuals was also enrolled. Patients with oncological and/or rheumatological and patients which have underwent recent orthopedic surgery and/or recent trauma with respect to the acquisition date were excluded from the study. A further exclusion criterion was established on the homogeneity of the data. In order to be included in the study, a patient had to perform the same exercises and had the same EMG recorded channels in the pre and post treatment assessments.

**Figure 1:**
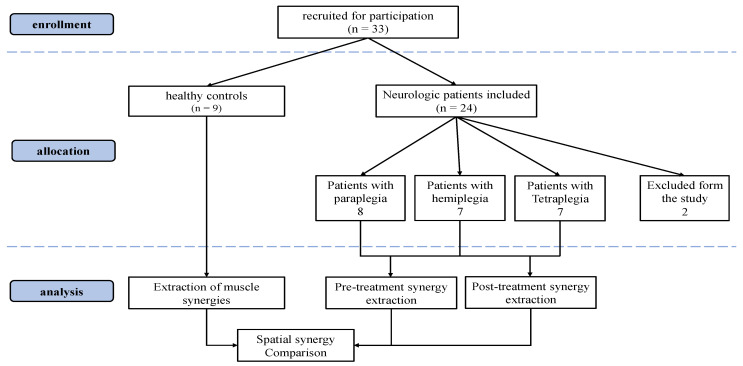
Consolidated Standards of Reporting Trials (CONSORT) flow diagram.

Two patients were excluded due to inhomogeneous muscle acquisitions between pre and post treatment sessions (at least one different EMG channel, or different executed exercises). The total number of subjects included in the analysis was 22 patients (7 hemiplegia, 7 paraplegia and 8 tetraplegia) and 9 healthy controls. In the CONSORT flow diagram (Figure 1), we illustrate the details of the enrollment procedure.

All patients underwent rehabilitation sessions at the VIKTOR Physio Lab^®^ physiotherapy center. The center independently sought the opinion of the competent Ethics Committee. Each patient (or legal representative) has given consent to the processing of data. The procedures were performed in accordance with ethical standards as set out by institutional and national committee and with the Helsinki Declaration of 1975, as revised in 2000 [50].

The data used in this retrospective study was collected during the period spanning from November 2018 to December 2020 in the VIKTOR Physio LAB (VIKTOR S.r.l., Milan, Italy). All enrolled patients underwent experimental recordings with a 16-channel surface electromyography (FreeEmg BTS, Milan, Italy) in order to monitor the level of motor functions over the course of the exercises.

The physiotherapy treatment was performed by three qualified physiotherapists. Medical supervision of the treatment was carried out by Dr. Viktor Terekhov and was performed using VIK16 Workstation (VIKTOR S.r.l., Milan, Italy).

#### Treatment and Device: VIK16 Workstation AFESK™

The rehabilitation treatment was carried out according to the VIKTOR method used with the AFESK™ technology (Adaptive Functional Electrical Stimulation Kinesitherapy).

**Figure 2:**
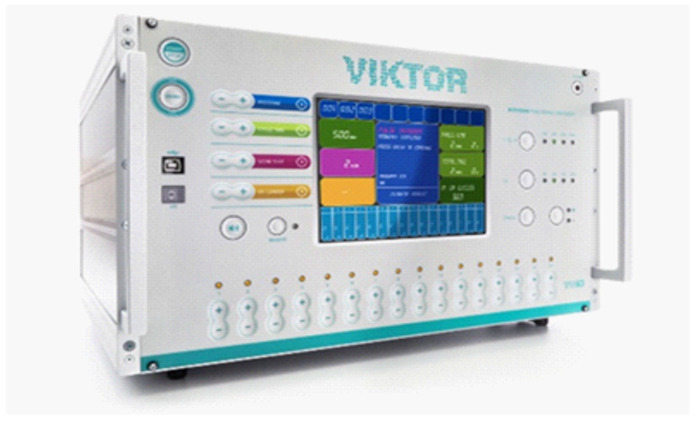

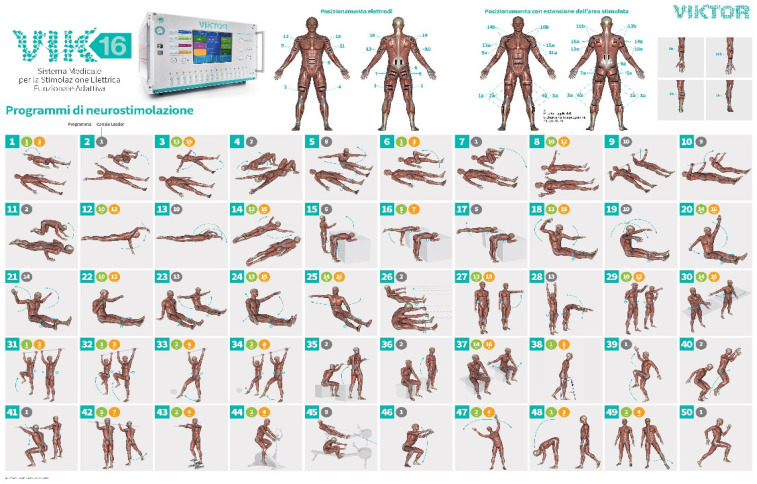
Graphic representation of the VIK16 Workstation and of the set of proposed total-body exercises. Workstation VIK16 has a library of 50 AFESK exercise programs that are used in rehabilitation, athletics and sports training. The programs are created on the basis of polymyographic and biomechanical assessment of the movement of healthy people, considering synergistic, reciprocal and antagonistic relationships of the moments of activation of the main muscle groups of the body. Workstation VIK16 has a wide range of electrical stimulation parameters: including current stabilized in each of 16 channels maximum of 150 mA, duration of a pulse from 100 to 1000 µs, pulse frequency from 50 to 200 Hz, motion cycle time from 200 ms to 10 s, impedance parameters and current level for each muscle group for all exercises performed by the patient, customizable number of cycles (movements) for each program and time for each exercise. In this study, only a subset of the exercises was performed by the enrolled patients.

The VIK16 Workstation technology has been developed exploiting the expertise achieved with more than thirty years of experience in using FES during exercise for the rehabilitation of neurological patients with severe lesions of locomotor functions. The Workstation VIK16 (Figure 2) is a device capable of supporting or partially replacing the CNS in the management of the motor scheme by delivering stimuli of suitable intensity to 16 muscles.

The method is based on percutaneous electrical stimulation of the neuromuscular system during cyclic exercises. For each muscle group involved in cyclic movements, electrical stimuli are given coinciding with the time activation in accordance with the physiological model of the exercise respecting the synergistic, reciprocal and antagonistic relationships between the muscles in each exercise.

In order to synchronize the patient’s movement with the supply of an electric stimulus to the muscle, during the exercise, a synchronized sensor or a sound signal were adopted, in order to trigger stimulation with the first muscle group moving during the selected program.

Thus, with the help of feedback control over the timely and correct performance, the motor function is implemented in the centers for motion control in the cerebral cortex. It is also documented that there is a rationalization of the efferent control of segmental mechanisms at the spinal level with the activation of vegetative support and sewerage of the afferent flow of information through the use of collateral interneuronal connections with adequate electrical excitation of the sensory receptor apparatus of the executive link (muscles, ligaments, joints, skin, etc.) [49,51,52,53,54,55,56,57].

When performing a cyclic movement, the electrical stimulation of the neuromuscular system uses movement as a system-forming function that combines the anatomical and physiological connections of the control system from segmental executive to cortical motion control centers [58]. At the same time, the muscle fibers of the muscle performing the cyclic movement are activated and the entire sensitive neuromuscular control complex of the segmental level, transmits afferent signals to the cortical centers of evaluation and movement control [59,60]. In pathology, the coordinated operation of some links in this chain can be disrupted by interrupting or changing the afferent flows of confidential afferent information; the electrical stimulation of peripheral afferents can alter the state of circuits not only within somatosensory cortex, but also within the motor network: It follows that whole body FES is of a multi-stage hierarchical process in which various elements of the cortical motor network are consistently engaged [58,61]. When receiving adequate sensitive information, the cortical centers of motion control begin to restore control of the lost functions by including in the process of reorganizing the compensatory pathological stereotype of movement into a normal one [59,60].

Since each movement is the result of coordinated descending central commands that control the underlying segmental reflex-tuned executive neuromuscular apparatus, the EMG activity of the muscles that implement the movement reflects the frequency-time and amplitude parameters of the activity of these muscles and the objective evaluation of their functional capabilities [62,63]. In the case of adequate electrical stimulation of these muscles, the entire sensory apparatus available in the muscle pool forms an afferent flow of information to the cortical centers, using reflex ascending functionally organized paths. [64,65,66,67,68]. At the end of each session, the workstation VIK16 automatically records in the download the results of work of each patient and treatment.

### 2.2. Data Acquisition

#### 2.2.1. Patients’ Protocol

**Figure 3:**
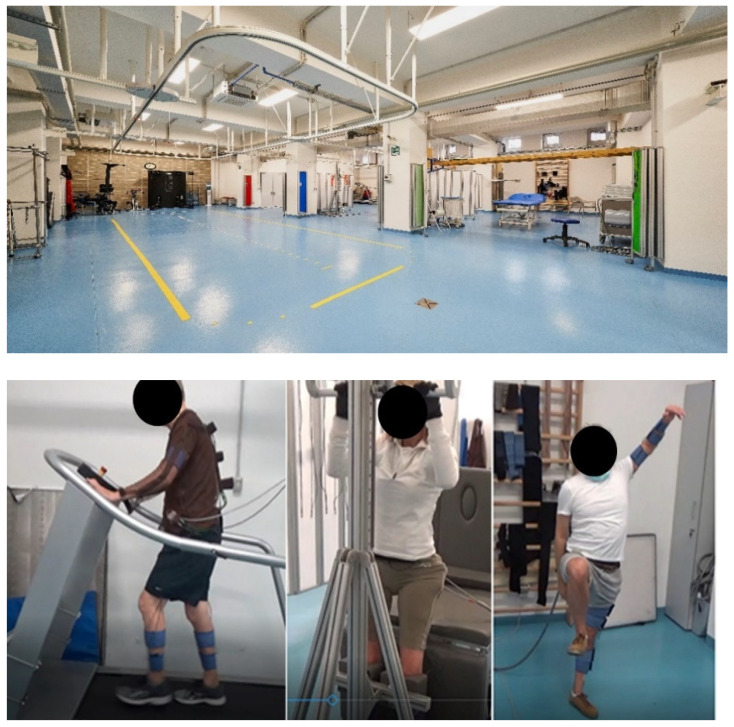
Employed set-ups for training at the VIKTOR Physio LAB.

The data was acquired in VIKTOR Physio Lab^®^ physiotherapy center (Figure 3). All patients underwent two instrumented acquisition sessions: before and after the treatment. In these two sessions, each patient was evaluated with the Arm, Trunk and Leg sections of the Motricity Index (MI). Each patient had his/her own customized FES treatment protocol and thus not all patients performed exactly the same exercises and had EMG recorded on the same muscles. However, the set-up and protocols were kept as homogeneous as possible across groups, compatibly with clinical needs. The muscles acquired were distributed on the whole body of the patient concentrating more on the impaired side of the body. Right hemiplegic patients had a denser EMG mapping on the right side of the body; left hemiplegic patients had more EMG sensors on the left emi-body; paraplegic and tetraplegic subjects were uniformly mapped on both body sides. All EMG probes were placed according to the SENIAM guidelines [69]. The acquired muscles changed between patient groups but were kept as homogeneous as possible in accordance with clinical needs and within patients of the same groups. The average age of participants was: Hemiplegia/paresis group: 52 years (not counting 1 child 6 years old); Paraplegia/paresis group—44 years; Tetraplegia/paresis group—46 years (not counting two children, 6 and 14 years old). The effective time of procedures in each session was on average 45 min.

The following program exercises were performed depending on the rehabilitation cycle:

(1) Introductory, adaptation: 7–10 exercises—on average 3–5 min each
(2) Restorative: 7–10 exercises—on average 3–5 min each
(3) Postural correctional: 5–7 exercises of which 1–2 last for 10–15 min
(4) Speed and endurance: 2–3 exercises of 15–20 min each
(5) Increase the duration of basic exercises: 3–5 exercises according to the program for 10–20 min each

Modality of electrical stimulation parameters were selected in accordance with the functional capability of each patient. Average values for each study group were listed in Table 1.

Before the beginning of the rehabilitation course, the sensitivity threshold of each muscle group was measured for each patient. The results obtained were used as reference for determining the level of current in the channels, which was supplied until the appearance of pronounced muscle contraction, without any pain. Usually, the values of the operating current, especially in patients with paresis, did not exceed twice the value of the sensitivity threshold. Stimulation parameters considered that the maximum permissible norms of current density during electrical procedures allow no more than 2 mA/cm^2^. Introductory and restorative exercises were performed at the beginning of the course, while postural, speed and endurance and increase of duration exercises were implemented with a proportional increase of time and speed of the exercise in order to increase the summation effects provided with AFESK on both sensory and motor links of neuromuscular regulation of motor functions. The average data for the performed treatments, including number of sessions, average movement per sessions and cycles are shown in Table 2.

During the period from November 2018 to December 2020, during which the rehabilitation of these patients was carried out, due to Covid-Sars 2, quarantine measures were repeatedly introduced with the closure of our center. For this reason, most patients, especially those with tetraplegia, reduced the number of visits, which reduced the average number of sessions for tetraplegic patients. In addition, all enrolled patients were in a stable chronic phase, after 2–10 years from the onset of the disease, and had already tried various methods of rehabilitation before admission to the center of the VIKTOR Physio LAB (VIKTOR S.r.l., Milan, Italy). They did not follow other rehabilitative treatments during the period of the FES training.

#### 2.2.2. Control Group Protocol

Healthy control subjects followed an acquisition protocol which encompassed all the set-ups employed with patients’ groups. The EMG recording protocol adopted for controls allowed to match muscles and exercises with all patients’ recordings. First, healthy controls performed the same exercises performed by patients. Given that previous studies confirmed that there is no major difference in muscle synergies for a wide variety of movements between the left and right limbs on healthy people [70], the muscles recorded on healthy controls were on the right hemi-body to match data for the hemiplegic, tetraplegic and paraplegic groups. Table 3 shows the muscles registered on patients and on healthy subjects to match the data of each patient group.

The exercises were a set of cyclical full body exercises expressively designed to perform active cyclical movements such as walking and specific movements to emphasize either upper-limbs, such as shoulder abduction, or lower-limb exercises, like knee adduction, or both in many cases. The set of the considered exercises could elicit many of the whole-body synergies available to subjects. All the exercises performed in the rehabilitation protocol are presented in Table 4.

### 2.3. Data Elaboration

**Figure 4:**
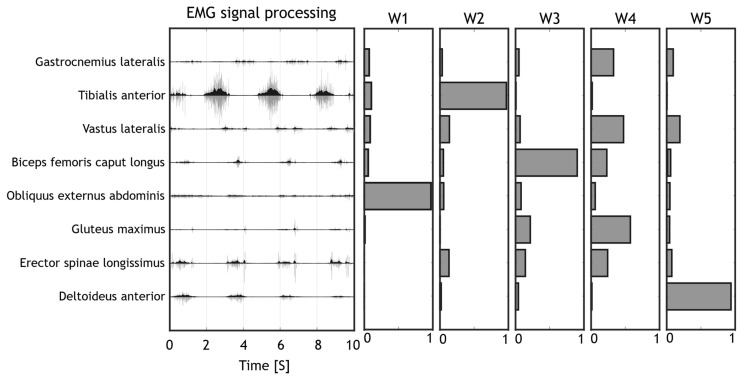
Pipeline for Signal processing. The raw signals (light grey) were filtered to remove movement artefacts and to compute the EMG envelope (dark grey). Muscle synergies were then extracted from the EMG envelope with the NMF algorithm.

The acquired EMG data was imported in MATLAB software (MathWorks, Natick, MA, USA) for the pre-processing. The EMG signals were filtered with a band-pass 6th order Butterworth filter covering a bandwidth from 30 Hz to 400 Hz, then they were full wave rectified, filtered with a low-pass 6th order Butterworth filter with cut-off frequency at 10 Hz, according to already employed processing pipelines for muscle synergies applications [71]. Lastly, the electromyographic data amplitude was normalized between zero and one to enable intra and inter subject comparisons, by dividing each channel EMG envelope by the maximum value found for that channel considering all movements performed by that subject in that session [72]. Time normalization was achieved by resampling each acquisition (EMG envelope) at 100 Hz. The elaborated data was organized in 2D arrays containing a concatenation of elaborated EMG data. Each column of the 2D array contained an EMG channel while each row contained the sequence of time samples. All exercises performed by the same subject were concatenated in the an array for the purpose of extracting synergies. A visual summary of the processing stage pipeline is provided in Figure 4.

### 2.4. Synergy Extraction

Muscle synergies were extracted from the elaborated EMG data using the non-negative matrix factorization algorithm (NMF) which is currently the most used algorithm for muscle synergy extraction. For our study, we used the spatial muscle synergy model, which extracts a set of spatial synergies containing muscle loads and a series of temporal coefficients indicating the time recruitment of each synergy. Synergies were extracted from each patient’s dataset, separating the pre-treatment and the post-treatment sessions, for a total of 2 sets of synergies per patient. The EMG electrodes and considered movements were the same for each patient in the two sessions. The number of extracted synergies was chosen by using the first order that reconstructed at least the 0.85 of the reconstruction R^2^ of the original signal [73].

#### Synergy Extraction: Control Group

Since patients from different groups had different EMG acquisition maps and different exercises routines, synergy extraction performed on the control group was repeated individually to match the data for each patient exercise routine and EMG mapping, by concatenating EMG from various repetitions and movements. The corresponding synergies from healthy controls were extracted only on the subset of the muscles and exercises specific for each patient. All healthy subject synergy sets were then averaged across controls and linked to the patient they refer to. Finally, each patient synergy set was ordered and compared to the corresponding healthy synergy set.

### 2.5. Outcome Measures

To compare synergies between healthy controls and patients, a synergy similarity metric was computed. The muscle synergy similarity (*SS*) is the dot product between two-unit norm synergies as shown in Equation (1).

(1) $$SS=W_{1}\;\cdot {\;W}_{2}$$

The synergy similarity metric was computed between matched couples of synergies between two sets of synergies (e.g., Hemiplegic patients before treatment and healthy subjects). Patients synergies were compared to healthy subjects’ synergies using *SS* both before and after treatment. Then, the mean *SS* (*mSS*) was computed and used as an indicator of the synergy performance of each patient with respect to healthy subjects.

### 2.6. Statistics

A statistical analysis was implemented in order to verify if after the treatment, the induced synergy modifications were significant. First, all distributions were tested for normality with the Kolmogorov-Smirnov test. Similarity distributions for each patient in both pre and post treatment followed a normal distribution. Pre and post treatment distributions for the Motricity Index was tested with a *t*-test. The significance level was set = 0.05. For muscle synergies, *mSS* were compared using a 1-way ANOVA test to assess if the treatment induced a modification in spatial muscle synergies. The ANOVA test was coupled with a post hoc Tukey-Kramer test. When submitting the retrospective study to the Ethical Committee, assuming a significance level of 0.05 and using a 1-way ANOVA test applied to the outcome variable for comparison, it was verified that with the available dataset, it was possible to obtain a level of statistical power above 0.8. This calculation was performed using GPower software [74].

## 3. Results

In this section, we first show the results of the treatment found with the Motricity Index (MI) in Table 5. Pre-post improvements were found for motor functions in many items of the motricity index. In Hemiplegic patients, arm MI (*p* < 0.0021) and leg MI (*p* < 0.0024) increased; no differences were found instead for trunk MI (*p* = 0.1723). Paraplegic patients’ arm and trunk had already full function at the beginning of the treatment and no change was found; leg MI improved (*p* < 0.0183). Tetraplegic patients’ arm MI and trunk MI did not improve (*p* = 0.0702, *p* = 0.0523, respectively); leg MI improved (*p* = 0.0446). For tetraplegic patients, all *p*-values are slightly lower or higher to the threshold for significance.

**Figure 5:**
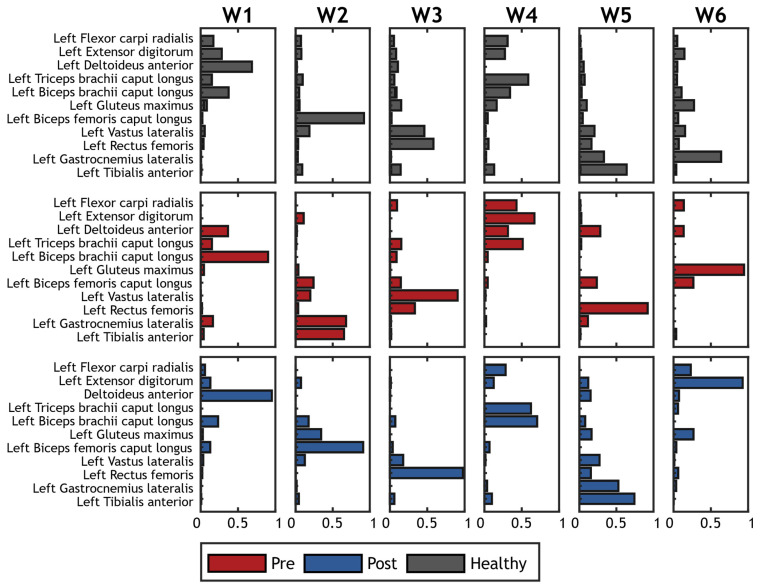
Example of synergies extracted on a hemiplegic patient. Spatial synergies before treatment are represented in red; spatial synergies after treatment are represented in blue. Grey bars show the corresponding reference synergies achived averaging synergies on the control group.

A typical example of the extracted synergies before and after treatment from a patient with hemiplegia is shown in Figure 5.

**Figure 6:**
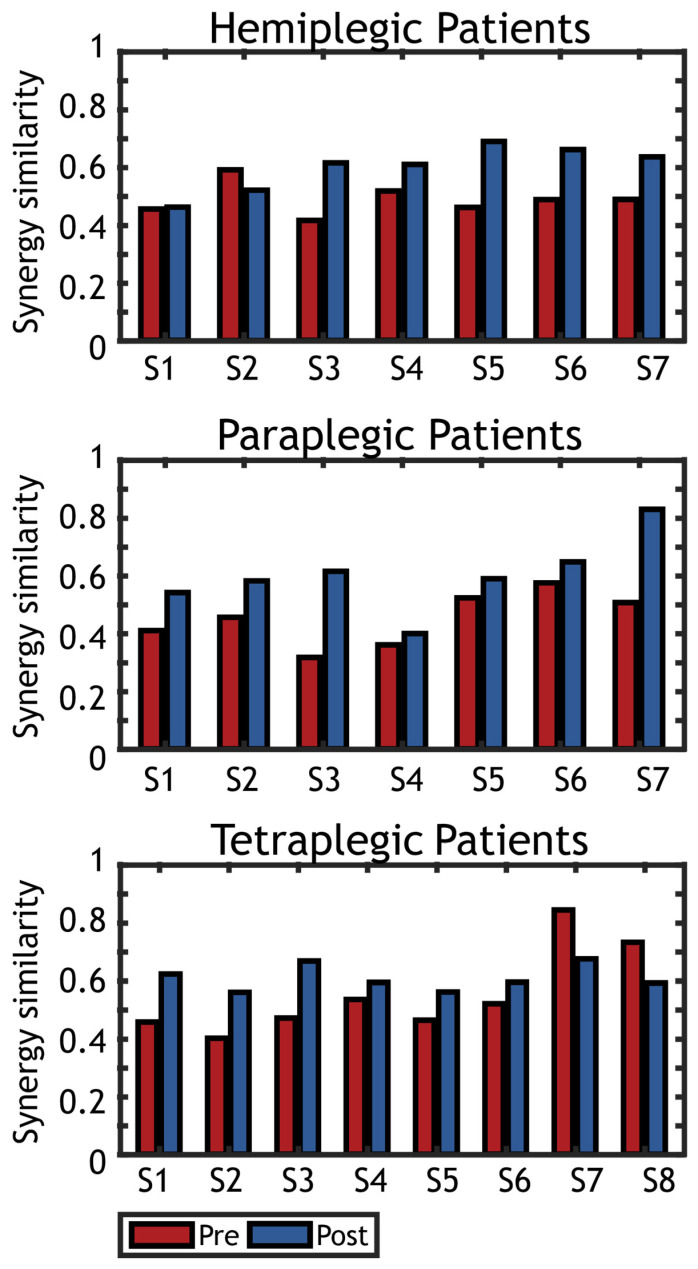
Spatial synergy similarity (healthy vs. tetraplegic) before (Pre) and after (Post) treatment. Graphs represent the similarity of the synergies extracted on each patient with the reference dataset of spatial synergies found on healthy controls. Pre-tratment synergy similarity is represented in red, while post-treatment synergy similarity is represented in blue.

The *mSS* obtained for all groups of subjects is presented in Figure 6.

**Figure 7:**
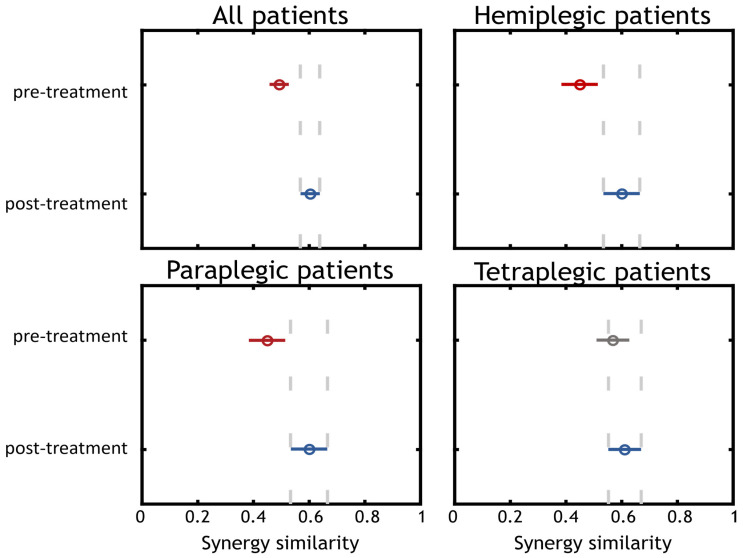
Statistical analysis. Statistical analysis was performed on each group of patients separately and for all subjects in the same group. We found that for the “All patients”, “Hemiplegic Patients”, “Paraplegic Patients” results were statistically significant (Post treatment synergy similarity in respect to controls increased), while for the “Tetraplegic Patients” group, there was a slight median increase of the MSS which was not statistically significant.

In Figure 7**,** the results of the statistical analysis are illustrated. The first panel shows the comparison between pre and post treatment for all patients. The other three panels illustrate the comparison for each group of patients separately.

The comparison including all patients showed a difference between pre and post treatment (*p* < 0.001). A median improvement was found increasing *mSS* from 0.50 in pre-treatment to 0.60 in post treatment. We also show the results achieved when dividing patients according to their disease. The results obtained from comparing pre and post trials for hemiplegic and paraplegic patients (*p* = 0.027 in both cases) showed an improvement in the synergy similarity from 0.45 to 0.60. The comparison between the pre and post trials for the tetraplegic group of patients did not yield a significant result (*p* = 0.454) but there was an improvement in the *mSS* from 0.57 to 0.61 (even if not significant).

## 4. Discussion

In this work, we have studied the effects of a total body AFESK treatment method on three groups of neurologic patients, composed of 22 neurologic patients: 7 hemiplegics patients, 7 paraplegic and 8 tetraplegic patients. They all underwent the same rehabilitation intervention protocol, aimed at restoring physiological muscle activation patterns by the means of total-body exercises coupled with multi-channel AFESK. This analysis describes one of the first attempts to combine whole-body FES with the muscle synergy assessment, a relevant biomarker for assessing inter-muscle coordination. Results are confirmed with clinical scales that also show motor improvements.

The results show the for most of the screened body segments, the Motricity Index increased after the treatment, indicating a partial recovery of the motor function.

The results also show a trend towards the restoring of healthy-like synergies was obtained, confirming previous findings achieved with local FES applications [44,75], and extending them to whole body approaches. Previous studies regarding muscle synergy analysis of FES based treatments only analyzed local FES applications, e.g., for walking [44] or for planar upper-limb movements [68].

Both studies have confirmed a tendency of subjects to re-align motor activation patterns to those of healthy subjects. This result is particularly meaningful because it was achieved in different pathologies and with chronic patients, during total-body functional movements strongly related with daily life activities.

Considering each group separately, only the paraplegic and hemiplegic patient groups achieved statistical significance; the tetraplegic group of patients showed also a slight improvement, even if not statistically significant. This result is most likely due to the lower number of sessions and the frequency of visits per week, as well as intervals between treatment sessions due to quarantine measures. We are also aware that this effect is probably related to the limited number of subjects included in the study. Interestingly, a slight improvement was seen both on clinical scales and with muscle synergies, but for both domains, results were mostly close to the threshold for statistical significance. These results should be confirmed on a higher number of subjects. At the same time, despite the fact that the time and frequency of stimulating effects in the tetraplegic group was lower than desirable for the maximum inclusion of reparation processes, positive changes in the level of muscle activity of the muscles were noted in most patients. In fact, while examining the group of patients as a whole, the results indicate a clear improvement in synergy similarity with the control group before and after the treatment.

At the diagnostic level, our results demonstrate the effectiveness of the whole-body FES approach and the appearance of changes at the local level of motor units. With further summation of the positive effects as a result of AFESK, a transition to more refined level of regulation can occur, in which the necessary levels of synergic interaction between the interested muscle groups will be more clearly manifested. The results obtained in this study indicate that whole body FES rehabilitation techniques could in fact be used to realign muscle activation patterns of neurologic patients to those of healthy people and promote neuroplasticity. The groups which benefited the most from the treatment were the group of paraplegic patients and the group of hemiplegic patients.

Despite muscle synergies can capture relevant aspects of muscular coordination patterns, they cannot fully describe the evolution of EMG patterns during the course of the therapy. In fact, for some patients, we did not observe significant changes in muscle synergy recruitment patterns, even though important modifications in clinical outcomes were observed with other methods (such as clinical scales, clinical tests, motor capability, and others).

One can observe that in four out of seven hemiplegic subjects, the treatment brought the synergistic muscular activity to a condition more similar with respect to the activations of the control group. On the contrary, in three patients, the treatment induced a change in the muscular activity, but this did not help the patients to restore muscle activation patterns closer to the control group.

All paraplegic patients underwent improvements in the activation patterns, although to a lesser extent it was expressed also in two patients whose period of injury that caused paraplegia exceeded 10 years, age—39 and 62 years, localization of damage—L2/3 and T 12-L2.

In the tetraplegic group of patients, five out of seven exhibited an improvement in the muscle activation patterns while only two could not. One of these cases had to interrupt the treatment in occasion of the birth of her child, after which the patient’s motor capabilities deteriorated, which was confirmed by the results of a repeated myographic examination. The second case is a patient with residual tetraparesis who completed a course of treatment after only 20 sessions.

Comparative myograms before and after the completion of the rehabilitation course of one of the patients with paraplegia level T 12-L2, who did not show modifications in synergic relationships, help clarify the effectiveness of the therapy which was not fully captured with muscle synergies. There was an increase in muscle activity of individual muscle groups, especially the rectus femoris, while walking with an exoskeleton. This result is not highlighted in muscle synergy analysis due to EMG normalization needed to compare synergies across subjects and sessions.

However, an in-depth analysis of changes in muscle activity also revealed a significant increase in the power spectrum of rapid motor units in the absence of significant changes in temporal activation parameters important for analyzing the synergistic relationships between muscle groups underlying movement (not reported here). Probably, the above case is an example of the accumulation of quantitatively functional changes in the neuromuscular apparatus, associated with an increase in the synchronization of the simultaneous inclusion of rapid motor units. The described effect can occur with an insufficient level of reflex regulatory influence on the part of the antagonists of their side, as well as the opposite side, which provides mutual reflex regulation with the participation of specialized interneurons of the segmental level.

With further repetition of AFESK movements according to this program, a further increase in the contractile capabilities of the muscle can occur, which can improve in the synergistic relationship between muscle groups that realize the movement.

Confirmation of the need for prolonged intensive exercises to restore lost functions were found in one of our patients with post-traumatic hemiplegia C1-2 level, who was excluded from the hemiplegia group due to the inconsistency of the protocol of the examined muscle groups that differed when comparing pre and post-therapy;. however, she managed to conduct a long course (190 sessions) with AFESK, including a high frequency of treatment (3–4 times a week), and time of movement execution and speed of movement constantly increasing. Currently, she can perform movement in full capacity.

### Limitations and Future Work

While this work provides clear evidence that total body FES helps restore physiological muscle coordination patterns, our results are affected by the low number of subjects involved in the study and non-homogeneous samples. Analyzing cohorts with small sample sizes could lead to non-conclusive results like in the case of the tetraplegic group of patients. Furthermore, non-homogeneity of the studied group should be avoided in future work.

Previous studies have confirmed the heterogeneity between different neurologic patients [44], which reinforces the need to have different protocols for different subjects. However, in order to provide reliable comparisons, a fully consistent protocol needs to be established. Despite this, due to the very low evidences available on total-body FES couples with muscle synergies, our study sets a relevant pilot work for more extensive applications in the future. We in fact noticed that research articles coupling muscle synergies and FES have high innovative approaches but always involve a very low number of subjects (from 2 to 9 patients) [41,42,44,76,77,78,79,80,81,82,83].

In addition to improved homogeneity of the cohorts, analyzing the improved performance of patients with only muscle synergies, one provides a deep, yet partial perspective on the actual quality of motion related to neurologic disorders. One effective way to overcome this limitation is with a conjunct analysis of both EMG and kinematics, for example by detecting the effects on kinematic and muscular patterns; this can be achieved with novel algorithms that allow inter-domain factorization [84]. Multi-domain approaches could be considered to enhance effect of rehabilitation and assessment [85,86].

In addition, given the experience of this study, it should be noted that there is a need for further development of the methodology for assessing synergic relationships of muscle activity in patients with severe neurological disorders of whole body and locomotor functions. The methods currently used in clinical practice do not allow to fully assess the functional nature of pathophysiological disorders of the whole body and locomotor apparatus. At the same time, the methodology used to assess the synergistic relationships of muscle activity during exercise can bring us closer to solving a multi-level assessment of violations in movement control. This was confirmed in our work by the coincidence of the results of the clinical evaluation of the state of patients with the conclusion made on the basis of synergic relationships for each of the interested muscle groups in patients with emi-para-tetraplegia.

If used, such a data collection system will allow to timely receive the necessary information about changes in locomotor functions in the process of rehabilitation timely change the tactics and set of rehabilitation programs, which in turn will certainly enhance the effect of therapy [87,88,89,90,91,92].

Lastly, it is interesting to evaluate the treatments capability to induce long term changes in patients. Thus, a follow-up session should be included in further studies on the topic.

## 5. Conclusions

In a few numbers of works, researchers have studied the possibility of either analyzing FES treatments with muscle synergies or using them for control of stimulation patterns. The studies that employed muscle synergies and FES, consistently reported positive outcomes in improvements in muscle synergies patterns for neurologic patients.

This work also adds to this pool of studies by reporting positive changes in patients which underwent whole body FES. It is necessary to be cautious when interpreting our results since more studies need to be performed on the matter and be guided by average indicators on a larger number of cases of the disease for each nosology. In addition, given the prospects of the direction of active whole-body FES in the rehabilitation of patients with severe neurological disorders, it is necessary to develop a comprehensive evaluation system considering clinical practice and objective research methods in the process of implementing locomotor functions.

## Figures and Tables

**Table 1:** FES parameters for hemiplegic patients.

Pulse Width (µs)/Pulse Frequency (Hz)			
Hemiplegia	200/100	after adaptation	500/50–100
Hemiparesis	100/50–100	after adaptation	200/100
Paraplegia	200/100	after adaptation	500/50–100
Paraparesis	100/50–100	after adaptation	200/100
Tetraplegia	200/100	after adaptation	500/50–100
Tetraparesis	200/50	after adaptation	200/100

**Table 2:** Rehabilitation treatment data (averages for hemiplegic, paraplegic and tetraplegic groups).

Hemiplegic	N° Sessions	Sessions/Week	Cycles Movements/Session	Total Movement Cycles
Hemiplegic	71	2.25	813	57,720
Paraplegic	80	2.25	705	56,461
Tetraplegic	40	1.4	667	26,709

**Table 3:** List of muscles acquired for the patients’ groups. The green coloured squares indicate which muscles were registered for each group. On healthy controls, EMG was placed on all muscles to match patients’ data.

	Hemiplegic Patients	Tetraplegic Patients	Paraplegic Patients
Biceps brachii caput longus			
Biceps femoris caput longus			
Deltoideus anterior			
Deltoideus posterior			
Erector spinae longissimus			
Extensor carpi radialis longus			
Extensor digitorum			
Flexor carpi radialis			
Gastrocnemius lateralis			
Gluteus maximus			
Latissimus Dorsi			
Obliquus externus abdominis			
Obliquus internus abdominis			
Rectus abdominis			
Rectus femoris			
Tibialis anterior			
Trapezius descendens			
Triceps brachii caput lateralis			
Triceps brachii caput longus			
Vastus lateralis			

**Table 4:** List of exercises performed by the patients’ groups. The green coloured squares indicate which exercises were registered for each group. Healthy controls performed all the exercises to match with patients’ data.

	Hemiplegic Patients	Tetraplegic Patients	Paraplegic Patients
Walking			
Crutch assisted walking			
Lying down arm and contralateral knee adduction			
Lying down Knees and arms abduction			
Lying down Jumping jacks			
Hip thrust			
Torso Torsion			
Knees adduction			
Prone to cat pose			
Sitting Punching			
Shoulder abduction			
Standing Punching			
Standing Knee adduction			
Sit to stand			
March			
Jump			
Squat			
Exoskeleton assisted movement			
Push			

**Table 5:** Motricity Index for Arm, Leg and Trunk in Hemiplegic patients, paraplegic patients and Tetraplegic patients.

Motricity Index		Hemiplegic Patients		Paraplegic Patients		Tetraplegic Patients	
Subject ID	Body Segment	PRE	POST	PRE	POST	PRE	POST
S1	ARM	10	34	100	100	40	73
	LEG	70	76	29	67	38	53
	TRUNK	25	25	100	100	61	74
S2	ARM	50	73	100	100	29	29
	LEG	91	100	76	100	1	1
	TRUNK	25	25	100	100	0	0
S3	ARM	34	41	100	100	29	29
	LEG	76	76	1	10	24	24
	TRUNK	25	25	61	61	0	0
S4	ARM	1	18	100	100	19	40
	LEG	24	29	28	28	19	39
	TRUNK	74	74	74	74	0	36
S5	ARM	10	15	100	100	29	29
	LEG	29	39	1	10	29	29
	TRUNK	100	100	74	74	24	29
S6	ARM	10	29	100	100	29	34
	LEG	19	29	1	10	29	29
	TRUNK	61	74	61	61	48	61
S7	ARM	19	29	100	100	92	100
	LEG	28	38	58	76	92	100
	TRUNK	61	74	87	87	100	100
S8	ARM					34	39
	LEG					38	53
	TRUNK					87	100

## Data Availability

Data are not available for privacy reasons.
